# Associations of environmental pollution with pro-oxidant, antioxidant and inflammatory markers in pregnant mothers and newborns

**DOI:** 10.3389/ftox.2025.1572486

**Published:** 2025-04-29

**Authors:** Antonin Ambroz, Jiri Klema, Andrea Rossnerova, Alena Milcova, Anna Pastorkova, Jana Pulkrabova, Ondrej Parizek, Veronika Gomersall, Tomas Gramblicka, Jaroslav Zelenka, Tomas Ruml, Nikola Vrzackova, Milos Veleminsky, Newroz Hasan, Jan Topinka, Radim J. Sram, Pavel Rossner

**Affiliations:** ^1^ Department of Toxicology and Molecular Epidemiology, Institute of Experimental Medicine CAS, Prague, Czechia; ^2^ Department of Computer Science, Faculty of Electrical Engineering, Czech Technical University in Prague, Prague, Czechia; ^3^ Department of Food Analysis and Nutrition, Faculty of Food and Biochemical Technology, University of Chemistry and Technology, Prague, Czechia; ^4^ Department of Biochemistry and Microbiology, Faculty of Food and Biochemical Technology, University of Chemistry and Technology, Prague, Czechia; ^5^ Faculty of Health and Social Sciences, University of South Bohemia, Ceske Budejovice, Czechia; ^6^ Department of Obstetrics and Gynaecology, Hospital Ceske Budejovice, Ceske Budejovice, Czechia; ^7^ Department of Obstetrics and Gynaecology, Hospital Karvina-Raj, Karvina, Czechia

**Keywords:** environmental pollution, maternal exposure to newborn, oxidative DNA damage, lipid peroxidation, antioxidant response and inflammatory cytokines

## Abstract

The aim of the study was to analyze the variables that modify the levels of oxidative DNA damage and lipid peroxidation in non-smoking mothers and their newborns from environmentally distinct localities of the Czech Republic: Ceske Budejovice (CB, an agricultural region) and Karvina (an industrial region). Personal, socio-economic and medical data, concentrations of particulate matter of aerodynamic diameter < 2.5 µm (PM2.5) and benzo[a]pyrene (B[a]P) in the ambient air, the activities of antioxidant mechanisms (superoxide dismutase, catalase, glutathione peroxidase) and antioxidant capacity), the levels of pro-inflammatory cytokines, the concentrations of persistent organic pollutants (POPs) in blood plasma/cord blood plasma and urinary levels of polycyclic aromatic hydrocarbons metabolites (OH-PAHs) were investigated as parameters potentially affecting the markers of DNA oxidation (8-oxo-7,8-dihydro-2′-deoxyguanosine, 8-oxodG) and lipid peroxidation (15-F_2_t-isoprostane, 15-F_2_t-IsoP). Significantly higher levels of POPs were detected in the plasma of mothers/newborns from CB (p < 0.001), while increased external levels of B[a]P and PM2.5, confirmed by analyzing urinary OH-PAHs, were found in Karvina subjects (p < 0.001). In mothers, multivariate analysis showed no significant difference in oxidative stress markers (15-F_2_t-IsoP, 8-oxodG) between the two localities. The analysis further revealed that neither in CB nor, unexpectedly, in Karvina, did PAH exposure affect maternal lipid peroxidation. Significant associations between OH-PAHs and 15-F_2_t-IsoP or 8-oxodG were observed only in newborns. In addition, multivariate analyses revealed a borderline significant association between locality and 8-oxodG in the urine of all newborns (p = 0.05). In conclusion, not only the maternal exposure of PAHs but also some POPs can negatively affect oxidative stress status in the early-life of newborns.

## 1 Introduction

Adverse environmental conditions during pregnancy can negatively impact maternal as well as child health (Desai et al., 2017). Due to their immature immune system and the differing activities of enzymes (xenobiotic biotransformation and antioxidant response), developing fetuses and early infants until the age of two are more sensitive to the harmful effects of exposure to toxic compounds (Khachatryan and Aleksandryan, 2006). The potential consequences of prenatal and early-life exposure depend on exposure timing, duration, and magnitude, but also on the interplay between the chemicals involved (Krausová et al., 2023). These exposures can result in molecular changes (modification of nuclear and mitochondrial DNA with 8-oxodG; shorter telomere lengths in newborns after maternal exposure) that pose a higher risk of some diseases during adolescence (Bongaerts et al., 2022) and may be critical for the increased incidence of diseases later in adulthood. Molecular alterations may lead to the reprogramming of the organism to adapt to the environmental conditions that occurred during prenatal development (Rossnerova et al., 2020). Although the placenta serves as a protective barrier against harmful compounds, it has been shown that many environmental contaminants [e.g., particulate matter (PM), persistent organic pollutants (POPs)] can cross it (Dugershaw et al., 2020). Indirect developmental effects are realized via environmental contaminants accumulated in the primary maternal tissue barriers at the site of entry exposure. These compounds might induce the release of inflammatory mediators and soluble signaling factors resulting in oxidative stress and inflammation (Dugershaw et al., 2020).

In the lower respiratory tract, exposure to ultrafine PM0.1 (PM of aerodynamic diameter <0.1 µm) and fine PM2.5 (PM of aerodynamic diameter <2.5 µm) particles can induce a non-specific immune response mediated by phagocytic alveolar macrophages which is linked with the release of pro-inflammatory and other mediators and the production of reactive oxygen species (ROS) (Feng et al., 2016). Maternal, systemic and placental oxidative stress can lead to macromolecular damage, such as DNA oxidation and lipid peroxidation (Johnson et al., 2021).

POPs represent a broad class of chemicals such as polycyclic aromatic hydrocarbons (PAHs), polychlorinated biphenyls (PCBs), organochlorine pesticides (OCPs), halogenated flame retardants, including novel brominated flame retardants (BFRs) and perfluoroalkyl substances (PFAS) (Parizek et., al 2023). PAHs, including benzo[a]pyrene (B[a]P), bound to PM are generated primarily during incomplete combustion of organic matter in the atmosphere. Yi et al. (2015) have reported that the transplacental PAH dose reaching the fetus is only 10% of that of the mother. Similar to PAHs, other aforementioned POPs may induce ROS generation, but the mechanism is not clear (Szilagyi et al., 2020; Ojo et al., 2021).

Oxidative stress is defined as an imbalance between pro-oxidant and antioxidant mechanisms induced by either increased ROS levels or insufficient antioxidant capacity. In general, ROS are necessary for proper cellular physiology and their essential level is beneficial for the regulation of the signaling pathways involved in cell growth, proliferation, and differentiation (Mittler, 2017). However, ROS can arise in response to various exogenous stimuli (e.g., exposure to environmental contaminants, diet), and their elevated levels can have deleterious effects for human health (Ruano et al., 2022). ROS overproduction is primarily limited by antioxidant enzymes [e.g., superoxide dismutase (SOD), catalase (CAT) and glutathione peroxidase (GPx)] (Pisoschi and Pop, 2015) and antioxidant non-enzymatic molecules mostly obtained from dietary intake (vitamin C and vitamin E, selenium) (Diaz-Garcia et al., 2022). However, if intrinsic antioxidant mechanisms are overwhelmed due to excessive ROS levels, transcription factors (NF-κB, AP-1) propagating the pro-inflammatory signaling cascade are activated, and cytokines (e.g., TNFα, IL-1β, IL-6), chemokines and other inflammatory molecules are released. Cell damage can be caused by an induction of inflammation and by excessive amounts of ROS that interact with cellular macromolecules (DNA, lipids, proteins) (Delfino et al., 2011). The urinary concentration of 8-oxo-7,8-dihydro-2′-deoxyguanosine (8-oxodG), the most commonly oxidized nucleoside of DNA, is an established biomarker of oxidatively damaged DNA following chemical exposure (Koppen et al., 2020). Isoprostanes, particularly 15-F_2_t -Isoprostane (15-F_2_t-IsoP), are considered to be the most reliable markers of lipid peroxidation *in vivo* (Milne et al., 2015). Oxidative stress is generally thought to be involved in the pathology of many pregnancy-related disorders (Wu et al., 2015), but basic oxidative stress levels are important for the proper development of the placenta (Ruano et al., 2022). The birth transition from an intrauterine hypoxic environment to an extrauterine neonatal environment that contains up to 5 times higher amounts of oxygen is associated with increased ROS production (Gitto et al., 2009). The newborn adaptation to postnatal conditions is associated with an increased susceptibility to oxidative stress. This vulnerability is linked not only with the effect of high concentrations of oxygen in the organism of a newborn but also with an underdeveloped capacity of immature antioxidant system, and a high concentration of free iron that catalyzes Fenton reaction converting H_2_O_2_ into highly toxic hydroxyl radicals (Perrone et al., 2019).

The current study is a follow-up to our previous study Ambroz et al. (2016), where we analyzed the effects of air pollution on “traditional” biomarkers (8-oxodG, 15-F_2_t IsoP) in newborns via maternal exposure. The results showed that air pollutants can affect oxidative damage in newborns and induce the adaptation of the adult organism to adverse environmental conditions. In the current study, we aimed to analyze additional parameters to provide a comprehensive description of oxidative stress status in non-smoking mothers and their newborns. Personal characteristics, concentrations of PM2.5 and B[a]P in the ambient air, activities of antioxidant mechanisms (enzymes: SOD, CAT, GPx, antioxidant capacities), levels of pro-inflammatory cytokines (TNF-α, IL-1β, IL-6) and the plasma levels of selected POPs, as well as urinary levels of PAH metabolites were investigated as parameters potentially affecting pro-oxidant and antioxidant processes influencing the levels of 8-oxodG and 15-F_2_t-IsoP.

## 2 Methodology

### 2.1 Study design

The study was designed to investigate independent factors potentially associated with lipid peroxidation or oxidative DNA damage in mothers and newborns. In mothers before delivery, the oxidation state was determined using 8-oxodG levels in urine. The analysis is non-invasive, convenient, and can be carried out without DNA isolation during which further oxidation can occur. Moreover, the analysis method was chosen so as not to cause any stress to the mother shortly before delivery. After delivery, as urinary 8-oxodG analysis is inappropriate due to contamination of urine with blood, maternal oxidative status was assessed using plasma IsoP levels. IsoP is generated by a plasma ROS attack on arachidonic acid and is enzymatically released from cell membranes to the bloodstream. Similar to urinary 8-oxodG formed by an oxidation of nucleotide pool (Chao et al., 2021), plasma IsoP levels are not affected by repair mechanisms. Both the plasma IsoP and urinary 8-oxodG level should reflect the overall oxidative status of an individual. Medical questionnaires provided information about mothers/newborns, the course of delivery (complications, procedures, medications, etc.) and the pregnancy outcome, and could be investigated for potential associations with oxidative damage. We hypothesized that in statistical models, PAH exposures will be better reflected on oxidative stress status compared to generally not metabolized POPs for which we expected limited effects.

### 2.2 Subjects and sampling

The cohorts originated from two geographically distant localities of the Czech Republic with specific local features: Ceske Budejovice (CB; a regional center in a mostly rural area characterized by fish farming, forestry and agriculture) and Karvina (a town in a region characterized by environmental pollution from heavy and mining industry). In each locality, 250 normal deliveries (38–41 weeks+) of non-smoking mothers and their newborns were included in the study. Written, informed consent was obtained from each mother, and their participation could be canceled at any time, according to the Helsinki II Declaration. The study was approved by the ethics committees of participating institutions: Institute of Experimental Medicine CAS in Prague, Ceske Budejovice hospital and Karvina hospital. The non-smoking mothers, recruited from June 2019 to August 2020, completed a personal questionnaire on medical history, socio-economic factors and lifestyle. Obstetricians provided information about the mother’s health during childbirth in a medical questionnaire. The data were collected continuously during summer 2019 (June–October), winter 2019/20 (November–April) and summer 2020 (May–August). Analyzed biological samples included venous blood/urine from mothers and cord blood/urine from newborns. Venous and cord blood were drawn to EDTA tubes to isolate plasma. Samples of plasma, used for analysis of antioxidant capacity (Oxygen Radical Absorbance Capacity; ORAC), antioxidant enzymes (SOD, CAT, GPx), pro-inflammatory cytokines (IL-1β; IL-6), selected POPs and 15-F_2_t-IsoP, were stored at −80°C. Urine samples were collected into 50 mL tubes (Greiner Bio-one Kremsmünster, Austria) and kept in aliquots at −80°C until analysis of creatinine, cotinine, PAH metabolites and 8-oxodG. Pairs with incomplete analyzed parameters were not included in the study. Finally, the number of pairs in the study was 147 in Karvina and 201 in CB.

### 2.3 Environmental pollution exposure monitoring

Information on the ambient air concentrations of PM2.5 and B[a]P (external exposure) during the sampling period was obtained from the database of the Czech Hydrometeorological Institute (CHMI; https://www.chmi.cz/?l=en). The study subjects lived within the range of the sampling sites. External exposure was measured by stationary monitors used by CHMI on the seventh day before delivery and collection of biological samples. Internal exposure to POPs and PAHs was performed by analysis of selected POPs and OH-PAH metabolites in plasma or urine after delivery.

### 2.4 Analysis of POPs in plasma

Targeted POPs included selected PCB congeners, some representatives of OCPs (dichlorodiphenyldichloroethylene, DDE; dichlorodiphenyldichloroethane, DDD; dichlorodiphenyltrichloroethane, DDT; hexachlorobenzene, HCB; hexachlorocyclohexanes, HCHs), BFRs (decabromodiphenyl ethers, BDE) and PFAS (perfluoro-1-butanesulfonate,PFBS; perfluoro-1-hexanesulfonate, PFHxS; perfluoro-1-octanesulfonate, PFOS; perfluoro-1-decanesulfonate, PFDS; perfluoro-n-butanoic acid, PFBA; perfluoro-n-heptanoic acid, PFHpA; perfluoro-n-octanoic acid, PFOA; perfluoro-n-nonanoic acid, PFNA; perfluoro-n-decanoic acid, PFDA; perfluoro-n-undecanoic acid, PFUdA; perfluoro-n-dodecanoic acid, PFDoA; perfluoro-n-tridecanoic acid, PFTrDA; perfluoro-n-tetradecanoic acid, PFTeDA). An analysis of the aforementioned plasma POPs was performed using gas chromatography coupled to (tandem) mass spectrometry [GC–MS/(MS)], and ultra - high performance liquid chromatography coupled to triple quadrupole tandem mass spectrometry (UHPLC–MS/MS), as previously described in detail (Polachova et al., 2021). The results were expressed in ng/mL plasma (for polar POPs) or ng/g of lipid weight (for non-polar compounds).

### 2.5 Creatinine assessment

To standardize the urine concentration in individual samples for data comparability, a creatinine assessment was performed. The determination of the creatinine level in the urine samples was analyzed using a Jaffe method based on the reaction with picric acid (Delanghe and Speeckaert, 2011). The samples were measured in technical duplicates at 490 nm, 50 µL sample/well, and creatinine concentrations were expressed in g/L.

### 2.6 Analyses of PAH metabolites in urine

OH-PAH metabolites (naphthalene-1-ol, 1-OH-NAP; naphthalene-2-ol, 2-OH-NAP; fluorene-2-ol, 2-OH-FLUO; phenanthrene-1-ol, 1-OH-PHEN; phenanthrene-2-ol, 2-OH-PHEN; phenanthrene-3-ol,3-OH-PHEN; phenanthrene-4-ol, 4-OH-PHEN; phenanthrene-9-ol, 9-OH-PHEN and pyrene-1-ol, 1-OH-PYR) were assessed in urine. First, urine samples were processed using liquid-liquid extraction (LLE) with clean-up using solid phase extraction (d-SPE). OH-PAH metabolites were assessed in adjusted urine samples using tandem mass spectrometry. More details on the analytical procedure (extraction, instrumental procedure, and validation) are described in publications of previous studies (Urbancova et al., 2020). The concentration of the compounds was normalized per creatinine content (ng/g creatinine).

### 2.7 Cotinine assay

Urinary cotinine was measured using an ELISA kit (OriGene, Rockville, MD). An analysis of each sample was carried out in technical duplicate using a 10 μL sample/well. Urinary cotinine levels were expressed in ng/mg creatinine. Mothers with cotinine concentrations of more than 500 ng/mg were considered active smokers and were not included in the study.

### 2.8 Analyses of antioxidant enzyme activities

#### 2.8.1 Superoxide dismutase activity

The SOD activity was analyzed using an indirect method employing superoxide generation from the xanthine/xanthine oxidase system, and detection of a color product formed from nitroblue tetrazolium (NBT), as an indicator of superoxide production. The method is based on the competition between SOD and NBT for superoxide. In short, the reaction solution was prepared by mixing 100 μM xanthine, 250 μM NBT, 100 μM EDTA, and a plasma sample diluted 100x in phosphate buffer. This mixture (80 μL) was added in triplicate into a 37°C tempered 96-well plate, and a reaction was initiated using 20 μL of diluted xanthine oxidase in phosphate buffer (final enzyme activity: 0.2 mU/mL). The kinetics of the reaction at 560 nm was monitored at 37°C for 1.5 h. A calibration curve of the enzyme activities was used to determine the activity of SOD. The results were expressed in U/mL.

#### 2.8.2 Catalase activity

This method is based on the ability of CAT to decompose hydrogen peroxide (H_2_O_2_). The excess H_2_O_2_ can form a complex with colorless ammonium heptamolybdate tetrahydrate to produce a yellow product. CAT activity is evaluated indirectly by calculating the concentration of H_2_O_2_ using the resulting absorbance of the yellow product. In brief, the ammonium heptamolybdate tetrahydrate was dissolved in 0.025 M sulfuric acid. The reaction mixture of H_2_O_2_ (50 mM solution in phosphate buffer) and of 100x diluted plasma was prepared, and a volume of 80 µL was added in triplicate into a 96-well plate and incubated for 1 h at 37°C. A visualization of the excess H_2_O_2_ was performed by the addition of 20 μL of a 4% molybdate solution. The absorbance was measured at 360 nm and a reference wavelength of 600 nm. A calibration curve was used to determine the plasma CAT activity. The results were expressed in U/mL.

#### 2.8.3 Glutathione peroxidase activity

Glutathione peroxidase (GPx) activity was determined by a method based on the conversion of reduced glutathione (GSH) to its oxidized form (GSSG) catalyzed by GPx. The reaction is in a cycle with the regenerating GSSG to GSH using glutathione reductase and NADPH. The decrease in NADPH absorbance is, therefore, directly proportional to GPx activity. The reaction was performed in 100x diluted plasma samples using a commercial kit (Trevigen, Gaithersburg, MD, United States), according to the manufacturer’s recommendations. The results were expressed in U/mL.

#### 2.8.4 Oxygen radical absorbance capacity (ORAC)

The ORAC method is based on measuring the oxidative degradation of a fluorescent probe (dipyridamole) after being mixed with an oxygen radical initiator 2,2′-azobis(2-methylpropionamidin)dihydrochloride (AAPH) generating ROS. In short, plasma samples were diluted in 2.5 µM dipyridamole and phosphate buffer to a final dilution of 100x. An adjusted sample (80 µL) was added in triplicate to a 96-well plate, and the initiation of the reaction was performed by 20 µL of 100 mM solution of AAPH. The fluorescence was measured for 2 h at an excitation wavelength of 415 nm, and an emission wavelength of 480 nm at 37°C. A calibration curve of Trolox, an analogue of vitamin E, was used to determine ORAC, expressed in µM Trolox Equivalent.

### 2.9 Analysis of cytokines

The selected pro-inflammatory cytokines (TNFα, IL-1β, IL-6) were analyzed in plasma using commercial ELISA kits (a kit for TNFα from R&D Systems, Minneapolis, MN, United States; a kit for IL-1β from Boosterbio, Pleasanton, CA, United States; a kit for IL-6 from BioLegend, San Diego, CA, United States) in accordance with protocols from the manufacturer. Concentrations of cytokines were expressed in pg/mL.

### 2.10 Analysis of oxidative damage markers

#### 2.10.1 8-oxodG ELISA

For analysis of urinary 8-oxodG, The Highly Sensitive 8-OhdG Check ELISA (JaICA, Shizuoka, Japan) based on an indirect competitive ELISA format was used according to the manufacturer’s instructions with some modifications. The process for determination of 8-oxodG level also involved a purification of urine samples by solid phase extraction (SPE) before the ELISA assay was started (Rossner et al., 2013). Briefly, purified and diluted urine samples in PBS (1:2) were pipetted (a 50 µL sample/well) in technical duplicate into a microtiter plate and were incubated with primary antibody (N45.1) at 4°C overnight. The next day, after performing additional steps as recommended by the manufacturer, an absorbance was measured with SpectraMax^®^iD3 (Molecular Devices, San Jose, CA, United States) at 450 nm, and urinary 8-oxodG levels were calculated and expressed as nmol 8-oxodG/mmol of creatinine.

#### 2.10.2 15-F_2_t-IsoP ELISA

For analysis of 15-F_2_t-IsoP in plasma, an 8-isoprostane ELISA kit from Cayman Chemical Company (Ann Arbor, MI, United States) based on a competitive ELISA format was used. Before the immunoassay was started, a hydrolysis, a purification using 8-Isoprostane Affinity Sorbent (Cayman, Ann Arbor, MI, United States), and evaporation of the elution solution was carried out in each sample (125 μL). Finally, samples were dissolved in ELISA buffer (330 μL), and analyzed in technical duplicates (50 μL sample/well). Absorbance was determined by SpectraMax®iD3 (Molecular Devices, San Jose, CA, United States) at 405 nm, and plasma 15-F_2_t-IsoP level was expressed as pg 15-F_2_t-IsoP/mL plasma (Rossner et al., 2008).

### 2.11 Statistical analysis

Descriptive statistical analysis was performed using GraphPad Prism version 9.3.1 for Windows, GraphPad Software (Boston, Massachusetts United States, www.graphpad.com). Differences in parameters between the studied localities were identified using the Mann-Whitney U Test and Z-test. A multivariate regression analysis was performed in the R environment. The relationship between independent and dependent variables was modeled using linear regression. The traditional least squares estimate of model coefficients was utilized, and the estimation method employed QR decomposition. This choice is numerically stable and generally recommended for most cases. To increase the robustness of the analysis with respect to outliers and collinearity, and to avoid difficulties in model interpretation, we conducted data preprocessing tailored to each model individually. Firstly, variables with low variance were excluded. Secondly, each group of conceptually tightly related and strongly correlated variables were represented by a single variable, following the recommendation of a domain expert. Next, all variables with a variance inflation factor higher than 5 were automatically removed from the model. Then, potential outliers were identified based on Cook’s distance. Samples with Cook’s distance values significantly larger than the mean were analyzed, and those deemed influential were removed after assessing their impact on the model using residual and leverage plots. Finally, the models were fitted and evaluated using metrics such as R-squared, adjusted R-squared, and the F-test p-value (Supplementary Table S1).

## 3 Results

### 3.1 Characteristics of the study subjects

The characteristics of mothers and their newborns enrolled in the study are shown in Tables 1, 2. The distributions of mothers’ age, their education and cotinine levels (a marker of tobacco smoke exposure) differed significantly between localities. Age and educational level were significantly higher in mothers from CB. The comparison did not reveal a significant difference between maternal pre-pregnancy BMI. The mothers involved in the study were selected as non-smokers, and the significant difference in the distribution of cotinine levels most likely reflects the effect of passive smoking for some mothers from Karvina (Supplementary Table S2). The distributions of gestation length, birth weight, Apgar score, and the sex of newborns did not differ between localities. A significant difference was noted only for newborn length (Table 2).

**TABLE 1:** Maternal characteristics, intake of vitamins, the parameters of antioxidant and immune response and oxidative stress markers.

Ceske Budejovice (N = 147)			Karvina (N = 201)		
Variable	Mean ± SD	Median (min, max)	Mean ± SD	Median (min, max)	P
Maternal characteristics - M-W test					
Maternal age	31.16 ± 4.41	31.00 (18.00, 42.00)	29.50 ± 5.11	28.00 (19.00, 43.00)	<0.001
BMI before pregnancy (kg/m2)	24.03 ± 4.41	23.24 (15.94, 41.78)	24.87 ± 5.19	23.66 (16.90, 50.47)	0.19
Education (N, %) - Z-test					
Lower Education Level	68 (46.3%)		126 (62.7%)		0.002
University	79 (53.7%)		75 (37.3%)		0.002
Vitamins (N, %) – Z-test					
Without	18 (12.24%)		68 (33.83%)		<0.001
Folic acid	101 (68.71%)		101 (50.25%)		<0.001
Iron^a^	19 (12.93%)		13 (6.47%)		0.05
Vitamin C^a^	5 (3.40%)		5 (2.49%)		0.63
Others	4 (2.72%)		14 (6.97%)		0.06
Antioxidant capacity (µM TE) – M-W test					
ORAC	5.43 ± 1.16	5.28 (2.65, 9.32)	5.23 ± 1.15	5.01 (1.92, 14.21)	0.05
Inflammatory markers (pg/mL) – M-W test					
TNFα	40.64 ± 79.36	0.00 (0.00, 250.00)	33.92 ± 70.93	0.00 (0.00, 250.00)	0.33
IL1β	102.25 ± 66.64	112.66 (0.00, 291.31)	126.28 ± 110.11	96.45 (0.00, 500)	0.46
IL6	14.14 ± 21.48	6.94 (0.00, 134.95)	45.07 ± 33.54	36.25 (0.86, 177.33)	<0.001
Oxidative stress markers: 8-oxodG (nmol/mmol creatinine) and IsoP (pg/mL) – M-W test					
8-oxodG before delivery	1.37 ± 0.66	1.21 (0.43, 4.83)	1.37 ± 0.68	1.18 (0.55, 4.39)	0.72
IsoP after delivery	111.83 ± 42.05	110.32 (16.98, 225.93)	98.33 ± 46.03	89.12 (18.79, 241.26)	<0.001

^a^ Used in a mixture with other supplements.

**TABLE 2:** Neonatal characteristics, Apgar score, parameters of antioxidant and immune response and oxidative stress markers.

Ceske Budejovice (N = 147)			Karvina (N = 201)		
Variable	Mean ± SD	Median (min, max)	Mean ± SD	Median (min, max)	P
Birth characteristics – M-W test					
Gestation (weeks)	39.24 ± 1.14	39.00 (37.00, 41.00)	39.33 ± 1.12	39.00 (36.00, 41.00)	0.34
Birth weight (g)	3475.8 ± 409.8	3490.00 (2320.0, 4370.0)	3408.2 ± 421.1	3360.0 (2340.0, 4830.0)	0.07
Birth length (cm)	50.13 ± 1.77	50.00 (45.00, 55.00)	49.53 ± 1.84	50.00 (44.00, 54.00)	0.002
Apgar score (N, %) – Z test					
10	126 (85.71%)		180 (89.55%)		0.28
9	17 (11.56%)		15 (7.46%)		0.20
8	3 (2.04%)		4 (1.99%)		0.98
7	1 (0.68%)		2 (1.00%)		0.74
Sex of newborn (N, %) – Z test					
Boy	77 (52.38%)		104 (51.74%)		0.90
Girl	70 (47.62%)		97 (48.26%)		0.90
Antioxidant capacity (µM TE) and enzymes (U/mL) – M-W test					
ORAC	5.50 ± 1.44	5.24 (2.86, 11.90)	4.11 ± 1.25	4.05 (1.43, 8.33)	<0.001
SOD	0.49 ± 0.33	0.39 (0.12, 2.33)	0.52 ± 0.28	0.47 (0.16, 2.15)	0.009
CAT	44.92 ± 3.18	46.10 (37.35, 50.21)	39.55 ± 3.59	38.33 (33.41, 52.55)	<0.001
GPX	148.45 ± 43.60	151.37 (50.86, 255.50)	82.55 ± 24.23	81.27 (20.10, 144.75)	<0.001
Oxidative stress markers:8-oxodG (nmol/mmol creatinine) and IsoP (pg/mL) – M-W test					
8-oxodG	3.94 ± 1.77	3.63 (1.35, 15.67)	4.87 ± 1.84	4.52 (1.38, 13.20)	<0.001
IsoP	97.15 ± 50.44	87.43 (13.90, 298.66)	88.85 ± 49.77	74.80 (12.23, 259.05)	0.08

### 3.2 Concentrations of contaminants

The maternal and neonatal exposures to contaminants are reported in Supplementary Tables S2, S3. Significantly increased ambient air levels of B[a]P and PM2.5 were found in Karvina (Supplementary Table S2). This fact was confirmed by analyzing urinary OH-PAH metabolites, markers of internal PAH exposure in the organism, both in mothers and their newborns. In contrast, the plasma levels of some POPs, especially PCBs, OCPs, and PFAS were significantly higher in mothers and their newborns from CB (Supplementary Tables S2, S3).

### 3.3 Antioxidant and immune response parameters

Tables 1, 2 also provide basic information on the parameters associated with antioxidant and immune response variables. The parameters of antioxidant activities differed between the regions. As shown in Table 1, the mothers from CB had a significantly higher total antioxidant capacity and vitamin intake than those from Karvina. Similar to mothers, a comparison between newborns from different localities revealed a significant increase of antioxidant capacity in newborns from CB. To better characterize the antioxidant properties in newborns, the levels of antioxidant enzymes (SOD, CAT, GPx) were investigated. For all the enzymes, significant differences were found between the groups from CB and Karvina. The most striking was the almost 2-times higher activity of glutathione peroxidase in newborns from CB (Table 2). The plasma levels of inflammatory markers TNF-α, IL-1β and IL-6 suggested that immune response in mothers may be related to the total antioxidant capacity. Mothers from Karvina with lower antioxidant capacity had significantly elevated IL-6 levels compared to mothers from CB (Table 1). This could be due to a PM2.5 and PAH stimulation of the inflammatory response accompanied by oxidative stress.

### 3.4 Oxidative biomarkers (15-F_2_t-IsoP; 8-oxodG): non-parametric and multivariate testing

#### 3.4.1 Maternal oxidative status characterized by 8-oxodG before delivery

In pregnant mothers before delivery, no significant differences in oxidative DNA damage were observed between the two localities (M-W test; p = 0.72; Table 1). Using the linear least squares method (ordinary least squares; OLS), a regression maternal 8-oxodG model that includes the investigated variables in both localities was constructed. The fit of the model was not significant (p = 0.40) indicating no effect of the variables on urinary 8-oxodG excretion. Similarly, regression maternal 8-oxodG models for each locality individually were constructed separately. Interestingly, the fits of the models were close to the borderline of significance (*p = 0.08* for CB, *p = 0.06* for Karvina) (Table 3).

**TABLE 3:** Maternal 8-oxodG models.

Variable	Estimate	SE	t	Pr(>|t|)	
Maternal 8-oxodG model for both localities (p = 0.40)					
Risk factors (Gilbert syndrome)	5.83E-01	1.57E-01	3.70	<0.001	***
OH-PHEN	1.29E-01	3.57E-02	3.60	<0.001	***
Maternal 8-oxodG model for CB (p = 0.08)					
Education	−2.19E-01	9.00E-02	−2.43	0.02	*
Risk factors (chronic)	9.88E-01	4.38E-01	2.26	0.03	*
Risk factors (Gilbert syndrome)	8.48E-01	2.83E-01	3.00	<0.001	**
Long term diagnosis (allergic)	6.00E-01	2.74E-01	2.19	0.03	*
Long term diagnosis (DM, hypertension)	−1.03E+00	5.73E-01	−1.80	0.08	.
Cotinine	−3.32E-02	1.43E-02	−2.32	0.02	*
PCB 28	2.54E-01	9.05E-02	2.80	0.01	**
β HCH	−1.95E-02	1.03E-02	−1.89	0.06	.
BDE 47	−7.71E-02	4.18E-02	−1.84	0.07	.
PFHxS	−2.02E+00	9.95E-01	−2.03	0.05	*
Maternal 8-oxodG model for Karvina (p = 0.06)					
Risk factors (Gilbert syndrome)	7.43E-01	2.02E-01	3.68	<0.001	***
Cotinine	8.74E-03	4.50E-03	1.94	0.05	*
ORAC	9.54E-02	3.79E-02	2.51	0.01	*
OH-PYR	−7.49E-01	2.88E-01	−2.60	0.01	*
PCB 28	−3.55E-01	1.37E-01	−2.60	0.01	*
BDE 153	6.63E-02	3.68E-02	1.80	0.07	.
PFDoA	−2.72E+01	1.17E+01	−2.32	0.02	*
OH-PHEN	2.04E-01	4.90E-02	4.16	<0.001	***

Designation for Pr(>|t|): *** <0.001, ** <0.01, * < 0.05 and. < 0.1.

#### 3.4.2 Maternal oxidative status characterized by 15-F_2_t-IsoP after delivery

In mothers *postpartum*, the M-W test revealed significantly increased IsoP levels in those from CB (p < 0.001). The F-test for the overall regression IsoP model including both localities, was significant (p = 0.01) (Table 4). While locality as a variable was not among significant predictors, positive associations were observed for PCB28 (p = 0.002) and BDE153 (p = 0.03); a negative association with a pharmacological intervention in the induction of delivery was also found (p = 0.01). Another regression maternal model constructed separately for CB was not significant (p = 0.46) (Table 4). The findings from a significant regression maternal IsoP model for Karvina (p = 0.01) revealed a positive association of lipid peroxidation with IL-1β (p = 0.05), cotinine (p = 0.01), PCB28 (p = 0.05), BDE153 (p = 0.02), PFTrDA (p = 0.002). Significant negative associations were observed for spasmolytic treatment (p = 0.01) and PFDoA (p = 0.002). A difference in lipid peroxidation levels between sampling periods was also found (p = 0.02).

**TABLE 4:** Maternal and neonatal IsoP models.

Variable	Estimate	SE	t	Pr(>|t|)		Estimate	SE	t	Pr(>|t|)	
Maternal IsoP model for both localities: p = 0.01						Neonatal IsoP model for both localities: p < 0.001				
Pharmacological induction	−3.15E+01	1.20E+01	−2.62	0.01	**					
Prostaglandin treat.	2.00E+01	1.10E+01	1.82	0.07	.					
Analgesic treat.	1.56E+01	8.91E+00	1.76	0.08	.					
Anesthesia (yes)						−1.28E+01	7.20E+00	−1.77	0.08	.
Maternal IL-6						−2.09E-01	9.83E-02	2.12	0.03	*
ORAC						3.79E+00	2.12E+00	1.79	0.07	.
SOD						−2.56E+01	9.82E+00	−2.61	0.01	**
Sampling period	−1.17E+01	6.45E+00	−1.81	0.07	.	−4.13E+01	6.93E+00	−5.96	<0.001	***
PCB 28	1.26E+01	3.96E+00	3.19	0.002	**					
BDE 153	4.89E+00	2.22E+00	2.20	0.03	*					
PFHxS	5.99E+01	3.44E+01	1.75	0.08	.					
OH-FLUO						−5.44E+01	3.03E+01	−1.79	0.07	.
OH-PYR						−1.73E+02	7.37E+01	−2.35	0.02	*
OH-PHEN						1.75E+01	6.56E+00	2.67	0.01	**
Maternal IsoP model for CB: p = 0.46						Neonatal IsoP model for CB: p = 0.06				
Sex of newborn (female)						−1.98E+01	1.09E+01	−1.81	0.07	.
Prostaglandin treat.	4.30E+01	2.17E+01	1.98	0.05	.					
ORAC	−1.09E+01	3.99E+00	−2.73	0.01	**					
p_p_DDE	1.38E-01	7.09E-02	1.94	0.06	.					
PCB 118						−1.53E+01	5.60E+00	−2.72	0.01	**
BDE 47	6.98E+00	2.92E+00	2.39	0.02	*					
PFHxS	2.49E+02	7.60E+01	3.28	0.002	**					
PFBA						1.14E+03	4.30E+02	2.65	0.01	**
External BaP (CHMI)						−1.12E+01	5.30E+00	−2.11	0.04	
Maternal IsoP model for Karvina: p = 0.01						Neonatal IsoP model for Karvina: p < 0.001				
Type of delivery (VEX)						5.51E+01	2.97E+01	1.86	0.07	.
Spasmolytic treat.	−2.54E+01	9.83E+00	−2.58	0.01	*	−2.06E+01	9.17E+00	−2.24	0.03	*
Long term diag. (allergic)						−2.02E+01	1.00E+01	−2.02	0.05	*
Maternal IL-1β	8.45E-02	4.21E-02	2.01	0.05	*	1.10E-01	4.65E-02	2.37	0.02	*
Maternal IL- 6						−2.20E-01	1.20E-01	−1.84	0.07	.
SOD						−2.68E+01	1.36E+01	−1.97	0.05	.
Sampling period	−2.10E+01	8.63E+00	−2.44	0.02	*	−4.31E+01	9.59E+00	−4.49	<0.001	***
Cotinine	4.32E-01	1.57E-01	2.76	0.01	*	6.51E-01	2.92E-01	2.23	0.03	*
PCB 28	1.77E+01	8.82E+00	2.01	0.05	*	−8.38E+00	3.55E+00	−2.36	0.02	*
PCB 153						1.67E+00	7.08E-01	2.36	0.02	*
PCB 180						−1.14E+00	6.03E-01	−1.90	0.06	.
BDE 153	5.62E+00	2.36E+00	2.39	0.02	*					
PFDoA	−2.10E+03	7.08E+02	−2.97	0.004	**					
PFTrDA	3.87E+03	1.23E+03	3.15	0.002	**					
OH-FLUO						−8.69E+01	3.42E+01	−2.54	0.01	*
OH-PYR						−1.63E+02	8.83E+01	−1.85	0.07	.
OH-PHEN						1.38E+01	7.71E+00	1.79	0.08	.

Designation for Pr(>|t|): *** <0.001, ** <0.01, * < 0.05 and . < 0.1.

#### 3.4.3 Neonatal oxidative status characterized by 8-oxodG

In the case of neonatal 8-oxodG, a significant difference between localities was found (M-W test; p < 0.001) with increased levels in Karvina. The overall regression neonatal 8-oxodG model including both localities (p < 0.001) indicated that locality itself is a borderline significant predictor of 8-oxodG urinary excretion (p = 0.0518; Table 5, rounded to 2 decimal points). Separate neonatal 8-oxodG models for CB (p = 0.002) and Karvina (p < 0.001) were also significant (Table 5). A comparison of the variables from the overall neonatal 8-oxodG model and the individual model for Karvina helped to identify significant predictors with the same effect for both models. The results suggested that long-term (others) and pregnancy (gestational DM) maternal diagnosis, as well as cytokine levels in mothers could affect neonatal 8-oxodG levels (Table 5). As for the antioxidant response, while the negative association of GPx and CAT was linked with 8-oxodG levels in the overall model for both localities, ORAC was identified as a parameter that is positively associated with urinary 8-oxodG in two models (overall for both localities; for Karvina). When the overall neonatal 8-oxodG and the individual model for CB were compared, the sampling period, gender, and newborn length were identified as parameters with comparable effects. The effects of contaminants were not consistent and differed between the 8-oxodG models. In the overall neonatal 8-oxodG model for both localities, PCB28 (p = 0.03) and PFDoA (p = 0.04) were positively associated with oxidative DNA damage. In the neonatal 8-oxodG model for CB, positive correlations of PFOA (p = 0.02) and cotinine (p = 0.007) were observed. In the neonatal 8-oxodG model for Karvina, a positive association with OH-NAP (p = 0.05) and a negative association with PFOS (p = 0.01) was found.

**TABLE 5:** Neonatal 8-oxodG models.

Variable	Estimate	SE	t	Pr(>|t|)	
Neonatal 8-oxodG model for both localities: p < 0.001					
Maternal medication (analgesic)	1.87E+00	1.11E+00	1.68	0.09	.
Maternal long-term diagnosis (others)	8.05E-01	3.31E-01	2.43	0.02	*
Pregnancy diagnosis (gestational DM)	1.19E+00	4.91E-01	2.41	0.02	*
Maternal IL-1β	−2.47E-03	1.18E-03	−2.11	0.04	*
Maternal IL-6	−9.46E-03	3.31E-03	−2.86	0.005	**
Locality (Karvina)	8.74E-01	4.47E-01	1.95	0.05	.
Sampling period	7.78E-01	2.44E-01	3.19	0.002	**
Sex of newborn (female)	9.16E-01	2.06E-01	4.46	<0.001	***
Newborn length	2.78E-01	8.17E-02	3.40	0.001	***
Neonatal SOD	−5.75E-01	3.32E-01	−1.74	0.08	.
Neonatal CAT	−7.83E-02	2.83E-02	−2.77	0.006	**
Neonatal GPX	−6.26E-03	2.85E-03	−2.19	0.03	*
Neonatal ORAC	1.27E-01	7.13E-02	1.78	0.08	.
PCB 28	1.91E-01	8.67E-02	2.20	0.03	*
PFDoA	3.39E+01	1.68E+01	2.02	0.04	*
PFOS	−3.89E-01	2.33E-01	−1.67	0.09	.
Neonatal 8-oxodG model for CB: p = 0.002					
Gestation (ultrasound)	4.45E-01	2.63E-01	1.70	0.09	.
Sex of newborn (female)	5.21E-01	2.84E-01	1.83	0.07	.
Birth weight	−8.94E-04	5.16E-04	−1.73	0.09	.
Newborn length	2.62E-01	1.18E-01	2.23	0.03	*
Sampling period	1.31E+00	4.81E-01	2.73	0.008	**
External BaP (CHMI)	4.16E-01	2.30E-01	1.81	0.07	.
OH-PYR	8.46E+00	4.36E+00	1.94	0.06	.
Cotinine	1.53E-02	5.53E-03	2.763	0.007	**
p,p-DDD	5.70E-02	3.40E-02	1.68	0.09	.
PFOA	2.20E+00	8.90E-01	2.47	0.02	*
PFHxS	−1.21E+01	6.40E+00	−1.88	0.06	.
Neonatal 8-oxodG model for Karvina: p < 0.001					
Maternal long-term diagnosis (others)	1.15E+00	4.84E-01	2.37	0.02	*
Pregnancy diagnosis (gestational DM)	2.23E+00	8.14E-01	2.74	0.007	**
Maternal IL-6	−9.36E-03	4.51E-03	−2.08	0.04	*
Sex of newborn (female)	1.37E+00	3.38E-01	4.05	<0.001	***
Neonatal ORAC	3.77E-01	1.21E-01	3.13	0.002	**
OH-NAP	5.00E-02	2.57E-02	1.95	0.05	*
PFOS	−1.01E+00	3.92E-01	−2.57	0.01	*

Designation for Pr(>|t|): *** <0.001, ** <0.01, * < 0.05 and. < 0.1.

#### 3.4.4 Neonatal oxidative status characterized by 15-F_2_t-IsoP

In newborns, the M-W test found no significant difference in lipid peroxidation between localities (*p = 0.08;* Table 2). However, the trend was similar to maternal lipid peroxidation and IsoP levels were higher in CB. In the significant overall regression neonatal IsoP model, including variables from both localities (p < 0.001), the locality was again not among the significant predictors (Table 4). The model further shows the negative association of neonatal lipid peroxidation with maternal IL-6 (p = 0.03) and neonatal SOD (p = 0.01). While 15-F_2_t-IsoP was positively associated with OH-PHEN (p = 0.01), for OH-PYR, a significant negative correlation was detected (p = 0.02). Furthermore, a difference in lipid peroxidation between sampling periods was found (p < 0.001). Similar to the maternal IsoP model for CB (p = 0.46) the regression neonatal IsoP model for CB was also not significant (p = 0.06) and was also only weakly related to IsoP (Table 4). The significant regression neonatal IsoP model for Karvina (p = 0.001) identified predictors that were also observed in the regression maternal IsoP model for Karvina (IL-1β, spasmolytic treatment, cotinine, sampling period). A significant association of PCB 28 with IsoP levels was also found in both newborns and mothers, but the associations were the opposite way round. Moreover, when compared with the maternal model neonatal IsoP levels were significantly associated with additional contaminants: OH-FLUO (p = 0.01) and PCB153 (p = 0.02). In the case of the antioxidant parameters, only neonatal SOD activity was negatively associated with lipid peroxidation (p = 0.05). The analysis may also indicate a significant influence of long-term diseases diagnosed in mothers (allergies, asthma) on IsoP levels in Karvina’s newborns.

## 4 Discussion

In mothers, multivariate analysis showed no significant difference in oxidative stress markers between the two localities, either before delivery (measured by 8-oxdG) or after delivery (measured by IsoP). Maternal 8-oxodG models (overall, CB, Karvina) were not significant and the analyzed variables predicted oxidative damage only very weakly. Therefore, the associations observed in the non-significant 8-oxodG and other non-significant models were not explained. The significant overall maternal IsoP model suggested a protective effect of pharmacological intervention in induction of delivery. As part of the intervention, associations of partial treatments (oxytocin, prostaglandin, analgesic, spasmolytic) were investigated. In Karvina, the administration of spasmolytics was associated with decreased maternal as well as neonatal lipid peroxidation. Spasmolytic and spasmoanalgetic mixtures administered during delivery facilitate dilatation of the cervix (Rohwer et al., 2013). By the shortening of the first labor period, the mother is not exposed to potential traumas resulting from a longer delivery that could be linked to increased lipid peroxidation. This was observed in Karvina´s mothers to whom spasmolytics were administered in 30% of cases when compared with CB mothers (p < 0.001; Supplementary Table S4). Thus, spasmolytic treatment demonstrates a significant antioxidant effect. Another association, between IsoP and IL-1β, suggests that mothers from Karvina could be more affected by inflammation linked with lipid peroxidation. This observation can be interpreted as an effect of the total exposome in mothers from Karvina resulting in inflammation that can also induce NF-κB, stress response and others cell processes. NF-κB regulates a large variety of target genes such as cytokines, affects aryl hydrocarbon receptor (AhR) activation and expression and CYP enzyme activity (Vogel et al., 2014). As in the overall maternal model for both localities, lipid peroxidation was significantly positively associated with PCB 28 and BDE 153 in the maternal IsoP model for Karvina. Moreover, plasma levels of PFTrDA and cotinine were also observed as positive predictors of lipid peroxidation in this group. Simple comparisons of contaminants showed a difference in the type of environmental pollution between the localities. Compared to CB, elevated levels of PAH metabolites were found in mothers from Karvina. Alternatively, increased levels of some PCBs, OCPs and PFASs were observed in CB mothers. The use of PCBs was widespread in industrial and commercial applications until it was banned due to environmental pollution. While PCB 138, PCB 153, and PCB 180 enter the human body mainly through contaminated food, PCB 28 is commonly found in polluted indoor air and airborne PCBs in urban environments. Moreover, PCB 28 has been found following occupational exposure (Randerath et al., 2024). Semi-volatile PCBs, particularly the lower-chlorinated congeners, can be continuously released from products that were manufactured before the ban (Egsmose et al., 2016) and can affect human health (Othman et al., 2022). In our significant maternal IsoP models, the positive association with PCB 28 can be explained by the fact that low chlorinated PCBs have a greater potential to cause oxidative stress. PCB 28 is more readily metabolized than high chlorinated PCBs and may form not only ROS via the metabolic pathway of PCB but also increase the oxidative stress potential by formation of downstream metabolites (Liu et al., 2020). In the case of some OCPs, their elevated plasma levels also reflect exposure of the historical burden to environmental pesticides in predominantly agricultural CB. In general, both PCBs and OCPs have not been used in the Czech Republic for decades and with their gradual reduction, the exposure of the population is also decreased. In the case of PFASs, as in PCBs and OCPs, the main sources of exposure are diet and drinking water. The increased PFAS concentrations in CB mothers could be related to their older age (Richterová et al., 2018) and corresponding longer periods of bioaccumulation. Our recent study could suggest that increased concentrations of PFAS may be connected with food intake in mothers from CB (Pavlikova et al., 2022). Both PAHs and the aforementioned POPs can induce ROS generation, but the mechanism of POP-induced oxidative stress is not clearly established. In the case of BDE 153, the positive association with maternal IsoP could be explained by the upregulation of *CYP1A1* and *CYP1B1* gene expression resulting in AhR agonistic effects (Kanaya et al., 2019). PFAS, such as PFTrDA, also induce ROS generation, but the mechanism is not clear. One of the proposed pathways is through the activation of PPARα (Szilagyi et al., 2020; Ojo et al., 2021). PFAS are known for their bioaccumulation, resulting from an extremely slow elimination rate and low biodegradability (Dickman and Aga, 2022). As a result, the organism can be exposed to the constant effects of these compounds for a very long time, even without their further intake. In the case of cotinine, non-smoking mothers participated in this study and the significant associations were attributed to the effect of passive smoking. Furthermore, although the external exposure to PAH confirmed by the internal levels of OH-PAH metabolites were significantly increased in Karvina mothers compared to CB, no associations between IsoP and OH-PAH were observed. This can be explained by the fact that PAH exposure did not reach the threshold to induce oxidative damage in mothers. However, in newborns, the same maternal PAH exposure affected oxidative damage.

In newborns, the total IsoP model showed that locality was not a significant predictor for lipid peroxidation. In the same model, a negative association was observed between neonatal lipid peroxidation and maternal IL-6. In adults, IL-6 also possesses anti-inflammatory effects through the synthesis of acute phase proteins. The cytokine can induce a systemic response to limit inflammation including the generation of ROS, leading to a reduction in oxidative damage. However, the synthesis of acute phase proteins is not yet developed in newborns and from a diagnostic clinical perspective, the result was insignificant, i.e., higher maternal IL-6 levels did not indicate higher neonatal lipid peroxidation. The negative association between lipid peroxidation and SOD may indicate sufficient elimination of an excessive production of superoxide in newborns. However, a marginally positive significant association was found for the total antioxidant capacity defined by all activities of the antioxidant components. This would mean that antioxidant mechanisms were activated, but their effect was insufficient to eliminate ROS. A simple comparison between the total maternal and neonatal IsoP models showed that significant associations with OH-PAHs were observed only in neonatal models, mainly in Karvina. This could be explained by the fact that newborns are more susceptible to oxidative stress than mothers and do not have developed regulatory mechanisms between the pro-oxidant and antioxidant processes leading to adaptation to adverse environmental conditions. In newborns, a positive association between OH-PHEN and 15-F_2_t-IsoP was observed; however, for other compounds (OH-PYR; OH-FLUO), significant negative correlations were detected. This can be explained by the fact that PAHs can act in a mixture; possible synergistic or antagonistic effects have been reported (WHO, 2010). In the case of POPs, transplacental transfer was also expected but significant associations of these compounds with lipid peroxidation were considered unexpected due to the low biodegradability and low metabolic activity. Indeed, the impacts of POPs on lipid peroxidation were observed only with PCBs compared to 8-oxodG neonatal models where associations were seen not only with PCBs but also mainly with PFASs. In the literature, both lipid peroxidation and DNA damage are associated with PCB exposure in adults (Liu et al., 2020). Due to the slow degradation of PCBs, it is unlikely that ROS would be formed via the metabolic pathway of newly presenting maternal PCBs in newborns. Oxidative damage may be caused by OH-metabolites of PCBs formed in the maternal organism and transported across the placenta (Park et al., 2009). This could be the explanation for the two opposite associations (positive with PCB 153, negative with PCB 28) with lipid peroxidation in newborns. In the neonatal IsoP model for Karvina, some of the significant predictors (IL-1β, spasmolytic treatment, cotinine, sampling period) were also found in mothers. This could be a confirmation of the effect of maternal exposure on newborns. The comparable results for IL-1β and cotinine suggested that neonatal lipid peroxidation was influenced by maternal inflammation and passive smoking. On the contrary, spasmolytic treatment has a protective effect on both mothers and newborns. Sufficient neonatal SOD enzyme activity may have played a role in the elimination of ROS produced during inflammation. In addition, IsoP levels in newborns from Karvina were associated both with concentrations of particular OH-PAH metabolites (OH-FLUO) detected in urine and some POP concentrations (PCB 28, PCB 153) observed in cord blood plasma. A finding of the negative association with the diagnosed maternal allergy in the neonatal IsoP model can seem unexpected. The questionnaires revealed 17% of mothers with allergies in Karvina, a significant difference compared to 7% of mothers from CB. However, fetal exposure via the mother’s organism can lead to the development of allergic diseases associated with oxidative stress later in life. In the development of all non-communicable diseases, such as allergies, many other factors also play a crucial role. Therefore, the individual consequences of the mother’s allergic disease on the newborn cannot be estimated based only on the diagnosis (Suh et al., 2017).

In newborns, the overall 8-oxodG model also revealed a significant association between oxidative DNA damage and locality. This was not observed in the neonatal lipid peroxidation models. This may be due to the different dynamics of oxidative stress marker production (England et al., 2000). Further, neonatal 8-oxodG models seem less influenced by the possible effects of medication administration as observed in the IsoP models. Using the overall model describing neonatal 8-oxodG levels from both localities, cord blood plasma levels of contaminants (PCB 28, PFDoA) were shown as the significant positive predictors for oxidative DNA damage. In the neonatal 8-oxodG model for CB, cord blood plasma levels of PFOA and cotinine positively correlated with 8-oxodG. On the contrary, in Karvina´s newborns, cord blood plasma levels of PFOS were in a negative association. Moreover, significantly increased oxidative DNA damage was positively associated with naphthalene (NAP) exposure. NAP is both a volatile organic compound and a PAH that is widespread both in indoor and outdoor air. Potential sources for humans include vehicle exhaust, evaporated gasoline, cigarette smoke, moth and pest repellants, toilet and diaper pail deodorizers, and air fresheners (Batterman et al., 2012). Jia and Batterman (2010) concluded that personal and residential concentrations are similar, while ambient concentrations are about an order of magnitude lower. Thus, it can be supposed that NAP exposure associated with increased neonatal oxidative DNA damage may be related to passive smoking or third-hand smoke (THS). The products of smoking can remain indoors and become reactivated long after smoking has ceased (Wu et al., 2022). In addition, the production of PFOS and PFOA was banned by the Stockholm Convention (POPS, 2024) but due to their properties they can still be toxic if present in the environment. Instead of PFASs, alternatives such as PFDoA, which differ in chain length, have been used commercially, and our study suggests that these substances can also be associated with oxidative DNA damage in newborns. Opposite associations with individual PFAS can be explained, as with PAHs, by possible synergistic and antagonistic/synergistic effects (Wee and Aris, 2023). In the overall neonatal 8-oxodG model, the activities of GPx and CAT were negatively linked with 8-oxodG levels, while in the neonatal 8-oxodG model for Karvina ORAC was identified as a borderline significant predictor positively associated with 8-oxodG, similarly as for the neonatal IsoP model. This would confirm that antioxidant mechanisms were activated in newborns, but their resulting effect was insufficient to eliminate ROS, particularly in Karvina. Other positive predictors that were found both in the overall 8-oxodG model and in the 8-oxodG model for Karvina were maternal long-term diagnosis (others), pregnancy diagnosis (gestational diabetes mellitus GDM) and sex of newborns (female). The group of maternal long-term diagnoses, identified here as “others”, consisted of various diagnoses that occurred with low frequency (thrombophilia; multiple sclerosis, Crohn’s disease, mental illnesses, etc.). The GDM is associated with a heightened level of oxidative stress in mothers (Lappas et al., 2011) which may also affect their newborns. The diagnostic process of GDM is still controversial (Amirian et al., 2020), which may be why no significant difference in GDM frequency was observed between localities in our study (Supplementary Table S5). The finding of a positive association between maternal GDM and 8-oxodG in Karvina´s newborns could indicate a higher number of GDM in Karvina´s mothers than was reported. This association was consistent with Castilla-Peon´s publication where it is mentioned that more oxidative DNA damage is induced in newborns of mothers with gestational diabetes (Castilla-Peon et al., 2019). In our study, the GDM may be confirmed by a higher level of maternal IL-6. This cytokine is an adept marker used in the diagnostic of GDM (Amirian et al., 2020) but multifunctional IL6 can also be expressed by a variety of cells after a multitude of stimuli. The negative association between maternal IL-6 and oxidative DNA damage in newborns may be explained by the study of Lappas et al. (2011). The authors reported that in the normal placenta, oxidative stress induces a significant increase in the expression and release of cytokines, an opposite effect that was observed in mothers with GDM. The authors explain this as a possible adaptation to protect the fetus from any further damage. Higher 8-oxodG levels were more often found in girls; this observation could be modulated by maternal exposome. A similar result was observed in our previous study Ambroz et al. (2016). However, the literature rather suggests that the male fetus should be more susceptible to intrauterine infections and other factors associated with oxidative stress (Ghosh et al., 2007).

## 5 Conclusion

In summary, maternal exposure in industrial locality was more strongly associated with higher PAH levels compared to the agricultural region. For maternal exposure to POPs, however, the levels of some substances were similar between both localities. The main source of exposure to these substances is diet, that could be similar in both localities due to shopping in the same supermarket chains in the Czech Republic. However, some levels of POPs (PCBs, OCPs and PFASs) were elevated in CB. In the case of OCP, this may also be affected by historical agricultural pesticide load. Due to the agricultural character, more food in CB can originate from local production and thus the dietary source can be different for both areas. Higher levels of PFAS in mothers from CB may be related to age and diet. Neither in CB, nor in Karvina, did exposure of PAHs affect lipid peroxidation in mothers. However, maternal exposure to PAHs had an effect on lipid peroxidation and oxidative DNA damage in newborns. Significant associations between OH-PAHs and IsoP were observed only in neonatal models, mainly in Karvina. Transplacental transfer of POPs was also confirmed, but findings of significant positive predictors (PFOA in 8-oxodG model for CB; PFDoA in overall 8-oxodG model) for oxidative DNA damage were considered unexpected given their properties. This fact is alarming due to the possible influence of PFAS on the oxidative status in newborns at such an early age. Our results showed that the total exposome of mothers in Karvina, which consisted of maternal inflammation, gestational DM, exposure to PAHs, but also PFAS, was more pronounced compared to CB. Together with the limited antioxidant defense in Karvina’s newborns, this contributed to a significant increase in oxidative DNA damage in this group.

## Data Availability

The raw data supporting the conclusions of this article will be made available by the authors, without undue reservation.

## Glossary

1-OH-NAP: naphthalene-1-ol
1-OH-PHEN: phenanthrene-1-ol
1-OH-PYR: pyrene-1-ol
2-OH-FLUO: fluorene-2-ol
2-OH-NAP: naphthalene-2-ol
2-OH-PHEN: phenanthrene-2-ol
3-OH-PHEN: phenanthrene-3-ol
4-OH-PHEN: phenanthrene-4-ol
9-OH-PHEN: phenanthrene-9-ol
8-oxodG: 8-oxo-7,8-dihydro-2′-deoxyguanosine
**15-F**_**2**_ **t-IsoP**: 15-F_2_t-isoprostane
AAPH: 2,2′-azobis(2-methylpropionamidin)dihydrochloride
B[a]P: benzo[a]pyrene
BDE: decabromodiphenyl ethers
BFRs: brominated flame retardants
DDD: dichlorodiphenyldichloroethane
DDE: dichlorodiphenyldichloroethylene
DDT: dichlorodiphenyltrichloroethane
GSH: reduced glutathione
GSSG: oxidized glutathione
HCB: hexachlorobenzene
HCHs: hexachlorocyclohexanes
NBT: nitroblue tetrazolium
OCPs: organochlorine pesticides
OLS: linear least squares method (ordinary least squares)
ORAC: Oxygen Radical Absorbance Capacity
PAHs: polycyclic aromatic hydrocarbons
PCBs: polychlorinated biphenyls
PFAS: perfluoroalkyl substances
PFBA: perfluoro-n-butanoic acid
PFBS: perfluoro-1-butanesulfonate
PFDA: perfluoro-n-decanoic acid
PFDoA: perfluoro-n-dodecanoic acid
PFDS: perfluoro-1-decanesulfonate
PFHpA: perfluoro-n-heptanoic acid
PFHxS: perfluoro-1-hexanesulfonate
PFOA: perfluoro-n-octanoic acid
PFOS: perfluoro-1-octanesulfonate
PFNA: perfluoro-n-nonanoic acid
PFTeDA: perfluoro-n-tetradecanoic acid
PFTrDA: perfluoro-n-tridecanoic acid
PFUdA: perfluoro-n-undecanoic acid
