# Correction: Associations of environmental pollution with pro-oxidant, antioxidant and inflammatory markers in pregnant mothers and newborns

**DOI:** 10.3389/ftox.2025.1650125

**Published:** 2025-07-04

**Authors:** Antonin Ambroz, Jiri Klema, Andrea Rossnerova, Alena Milcova, Anna Pastorkova, Jana Pulkrabova, Ondrej Parizek, Veronika Gomersall, Tomas Gramblicka, Jaroslav Zelenka, Tomas Ruml, Nikola Vrzackova, Milos Veleminsky, Newroz Hasan, Jan Topinka, Radim J. Sram, Pavel Rossner

**Affiliations:** ^1^ Department of Toxicology and Molecular Epidemiology, Institute of Experimental Medicine CAS, Prague, Czechia; ^2^ Department of Computer Science, Faculty of Electrical Engineering, Czech Technical University in Prague, Prague, Czechia; ^3^ Department of Food Analysis and Nutrition, Faculty of Food and Biochemical Technology, University of Chemistry and Technology, Prague, Czechia; ^4^ Department of Biochemistry and Microbiology, Faculty of Food and Biochemical Technology, University of Chemistry and Technology, Prague, Czechia; ^5^ Faculty of Health and Social Sciences, University of South Bohemia, Ceske Budejovice, Czechia; ^6^ Department of Obstetrics and Gynaecology, Hospital Ceske Budejovice, Ceske Budejovice, Czechia; ^7^ Department of Obstetrics and Gynaecology, Hospital Karvina-Raj, Karvina, Czechia

**Keywords:** environmental pollution, maternal exposure to newborn, oxidative DNA damage, lipid peroxidation, antioxidant response and inflammatory cytokines

The funder, “project National Institute for Cancer Research (Programme EXCELES - Funded by the European Union – Next Generation EU supported this work, ID Project No. LX22NPO5102)” was erroneously omitted.

The authors apologize for this error and state that this does not change the scientific conclusions of the article in any way. The original article has been updated.

