# Supplementary material for: Associations of environmental pollution with pro-oxidant, antioxidant and inflammatory markers in pregnant mothers and newborns
Source: Front Toxicol. 2025 Apr 29;7:1572486. doi: 10.3389/ftox.2025.1572486 (PMC12069330; doi:10.3389/ftox.2025.1572486)
Supplement: Supplementary file 1 [file Table1.docx]

Supplementary Material

| **Table S1** Model summaries | | | | | | | |
| --- | --- | --- | --- | --- | --- | --- | --- |
| Model | Residual standard error | Multiple R^2^ | Adjusted R^2^ | F stats | Degrees of freedom - residual | Degrees of freedom  - model | P |
| Maternal 8-xodG  both localities | 0.67 | 0.21 | 0.01 | 1.04 | 274 | 72 | 0.40 |
| Maternal 8-oxodG  CB | 0.60 | 0.54 | 0.15 | 1.40 | 78 | 66 | 0.08 |
| Maternal 8-oxodG  Karvina | 0.65 | 0.42 | 0.11 | 1.37 | 130 | 68 | 0.06 |
| Maternal IsoP  both localities | 42.76 | 0.28 | 0.09 | 1.48 | 274 | 72 | 0.01 |
| Maternal IsoP  CB | 40.73 | 0.48 | 0.01 | 1.03 | 74 | 67 | 0.46 |
| Maternal IsoP  Karvina | 42.14 | 0.45 | 0.16 | 1.58 | 131 | 68 | 0.01 |
| Neonatal IsoP  both localities | 45.09 | 0.34 | 0.16 | 1.88 | 268 | 72 | <0.001 |
| neonatal IsoP  CB | 46.15 | 0.57 | 0.18 | 1.45 | 74 | 69 | 0.06 |
| Neonatal IsoP  Karvina | 41.87 | 0.52 | 0.25 | 1.93 | 123 | 70 | <0.001 |
| Neonatal 8-oxodG  both localities | 1.52 | 0.41 | 0.26 | 2.66 | 271 | 72 | <0.001 |
| Neonatal 8-oxodG  CB | 1.18 | 0.67 | 0.34 | 2.02 | 70 | 69 | 0.002 |
| Neonatal 8-oxodG  Karvina | 1.60 | 0.51 | 0.25 | 1.92 | 127 | 70 | <0.001 |

| **Table S2** Levels of plasma POPs and urinary PAH metabolites in mothers from CB and Karvina | | | | | |
| --- | --- | --- | --- | --- | --- |
|  | Ceske Budejovice (N=147) | | Karvina (N=201) | |  |
| Variable | Mean ± SD | Median (min, max) | Mean ± SD | Median (min, max) | P |
|  | External Exposure | | | |  |
| PM2.5 (μg/m3) | 11.57 ± 9.03 | 9.20 (2.10, 51.10) | 22.35 ± 16.46 | 18.10 (0.00, 131.8) | <0.001 |
| B[a]P (ng/m3) | 0.66 ± 1.12 | 0.20 (0.00, 9.50) | 2.95 ± 7.80 | 0.90 (0.00, 74.80) | <0.001 |
|  | Internal Exposure | | | |  |
|  | Polychlorinated biphenyls (ng/g lipid weight) | | | |  |
| PCB 28 | 0.51 ± 0.82 | 0.21 (0.05, 4.64) | 0.50 ± 0.56 | 0.37 (0.05, 4.26) | 0.03 |
| PCB 118 | 3.81 ± 5.01 | 3.07 (0.05, 52.44) | 2.23 ± 1.87 | 1.82 (0.05, 13.37) | <0.001 |
| PCB 138 | 51.11 ± 193.72 | 22.90 (0.70, 2284.79) | 22.38 ± 29.12 | 15.14 (0.05, 305.68) | <0.001 |
| PCB 153 | 66.35 ± 207.94 | 37.15 (0.05, 2470.53) | 33.75 ± 45.36 | 22.05 (0.05, 493.16) | <0.001 |
| PCB 170 | 22.04 ± 41.79 | 14.18 (0.25, 470.25) | 18.07 ± 23.23 | 11.95 (0.25, 200.48) | 0.02 |
| PCB 180 | 45.71 ± 74.98 | 32.94 (0.25, 841.92) | 36.99 ± 47.93 | 25.12 (0.25, 472.02) | 0.002 |
|  | Organochlorinated pesticides (ng/g lipid weight) | | | |  |
| p,p'-DDE | 85.92 ± 80.77 | 67.53 (9.07, 644.93) | 78.33 ± 63.34 | 62.57 (16.60, 550.48) | 0.40 |
| p,p'-DDD | 1.76 ± 5.98 | 0.05 (0.05, 53.26) | 0.52 ± 0.91 | 0.05 (0.05, 6.12) | 0.04 |
| p,p'-DDT | 0.57 ± 1.29 | 0.25 (0.25, 9.10) | 5.31 ± 9.65 | 0.25 (0.25, 49.48) | <0.001 |
| HCB | 20.14 ± 28.32 | 8.81 (1.15, 150.25) | 8.82 ± 6.94 | 7.10 (1.84, 73.75) | 0.006 |
| β-HCH | 4.48 ± 6.90 | 2.99 (0.05, 45.34) | 0.80 ± 1.72 | 0.05 (0.05, 19.38) | <0.001 |
| γ-HCH | 1.39 ± 5.23 | 0.05 (0.05, 43.08) | Not detected | |  |
|  | Brominated flame retardants (ng/g lipid weight) | | | |  |
| BDE 47 | 1.42 ± 1.63 | 1.05 (0.05, 10.51) | 0.25 ± 0.60 | 0.05 (0.05, 7.39) | <0.001 |
| BDE 99 | 0.45 ± 2.20 | 0.15 (0.15, 20.27) | 0.30 ± 0.49 | 0.15 (0.15, 5.33) | 0.003 |
| BDE 100 | 0.22 ± 0.52 | 0.15 (0.15, 4.96) | 0.25 ± 0.39 | 0.15 (0.15, 4.33) | 0.02 |
| BDE 153 | 0.23 ± 0.49 | 0.15 (0.15, 4.53) | 0.41 ± 2.04 | 0.15 (0.15, 27.29) | 0.002 |
| BDE 209 | 1.98 ± 6.62 | 0.75 (0.75, 59.25) | 4.30 ± 23.15 | 0.75 (0.75, 220.28) | 0.98 |
| α-HBCD | Not detected | | 13.93 ± 161.50 | 0.25 (0.25, 2285.55) |  |
| TBBPA | Not detected | | 36.98 ± 504.96 | 0.20 (0.20, 7157.34) |  |
|  | Per- and polyfluoroalkylated substances (ng/mL plasma) | | | |  |
| PFHxS | 0.15 ± 0.09 | 0.13 (0.02, 0.65) | 0.16 ± 0.09 | 0.15 (0.03, 0.78) | 0.04 |
| PFOS | 1.28 ± 0.72 | 1.15 (0.21, 4.56) | 0.90 ± 0.63 | 0.74 (0.12, 5.13) | <0.001 |
| PFBA | 0.02 ± 0.01 | 0.02 (0.01, 0.04) | 0.03 ± 0.28 | 0.01 (0.01, 3.96) | <0.001 |
| PFHxA | 0.009 ± 0.008 | 0.005 (0.005, 0.073) | 0.005 ± 0.001 | 0.005 (0.005, 0.015) | <0.001 |
| PFHpA | 0.016 ± 0.017 | 0.012 (0.005, 0.178) | 0.009 ± 0.010 | 0.005 (0.005, 0.111) | <0.001 |
| PFOA | 0.60 ± 0.35 | 0.52 (0.10, 2.32) | 0.40 ± 0.24 | 0.35 (0.09, 1.84) | <0.001 |
| PFNA | 0.38 ± 0.32 | 0.31 (0.06, 2.76) | 0.13 ± 0.07 | 0.11 (0.03, 0.48) | <0.001 |
| PFDA | 0.16 ± 0.11 | 0.14 (0.03, 0.61) | 0.09 ± 0.06 | 0.07 (0.02, 0.40) | <0.001 |
| PFUdA | 0.07 ± 0.04 | 0.06 (0.01, 0.23) | 0.05 ± 0.03 | 0.04 (0.01, 0.22) | <0.001 |
| PFDoA | 0.02 ± 0.02 | 0.02 (0.01, 0.17) | 0.01 ± 0.01 | 0.01 (0.01, 0.06) | <0.001 |
| PFTrDA | 0.009 ± 0.008 | 0.005 (0.005, 0.069) | 0.008 ± 0.005 | 0.005 (0.005, 0.032) | 0.07 |
| Monohydroxylated PAH metabolites (µg/g creatinine) and cotinine (ng/mg creatinine) | | | | | |
| Σ OH-NAP | 10.93 ± 11.22 | 6.87 (1.46, 74.70) | 11.76 ± 10.06 | 8.54 (1.79, 56.31) | 0.05 |
| 2-OH-FLUO | 0.27 ± 0.21 | 0.23 (0.00, 2.13) | 0.50 ± 0.45 | 0.37 (0.11, 4.34) | <0.001 |
| Σ OH-PHEN | 0.82 ± 1.27 | 0.53 (0.17, 13.73) | 1.43 ± 1.29 | 0.98 (0.17, 10.45) | <0.001 |
| 1-OH–PYR | 0.09 ± 0.09 | 0.06 (0.02, 0.99) | 0.14 ± 0.22 | 0.09 (0.00, 2.10) | <0.001 |
| cotinine | 3.33 ± 22.33 | 0.00 (0.00, 264.55) | 4.91 ± 22.37 | 0.00 (0.00, 261.55) | <0.001 |

| **Table S3** Levels of blood cord plasma POPs and urinary PAH metabolites in newborns from CB and Karvina | | | | | |
| --- | --- | --- | --- | --- | --- |
|  | Ceske Budejovice (N=147) | | Karvina (N=201) | |  |
| Variable | Mean ± SD | Median (min, max) | Mean ± SD | Median (min, max) | P |
|  | Polychlorinated biphenyls (ng/g lipid weight) | | | |  |
| PCB 28 | 0.49 ± 1.10 | 0.05 (0.05, 6.97) | 0.24 ± 1.05 | 0.05 (0.05, 9.94) | <0.001 |
| PCB 118 | 0.93 ± 3.43 | 0.05 (0.05, 39.78) | 0.54 ± 1.44 | 0.05 (0.05, 10.09) | 0.12 |
| PCB 138 | 23.57 ± 42.37 | 12.51 (0.05, 321.48) | 12.03 ± 15.07 | 8.46 (0.05, 141.17) | <0.001 |
| PCB 153 | 26.27 ± 30.58 | 20.38 (2.87, 245.76) | 17.48 ± 19.34 | 11.88 (0.05, 179.70) | <0.001 |
| PCB 170 | 11.37 ± 10.12 | 9.34 (0.25, 68.45) | 5.49 ± 9.97 | 2.54 (0.25, 82.83) | <0.001 |
| PCB 180 | 26.00 ± 21.46 | 21.26 (2.28, 135.89) | 14.98 ± 20.63 | 9.41 (0.25, 179.99) | <0.001 |
|  | Organochlorinated pesticides (ng/g lipid weight) | | | |  |
| p,p'-DDE | 63.66 ± 47.25 | 55.93 (5.19, 446.32) | 49.31 ± 30.25 | 40.63 (0.05, 197.1) | <0.001 |
| p,p'-DDD | 1.00 ± 4.21 | 0.05 (0.05, 43.45) | 0.08 ± 0.47 | 0.05 (0.05, 6.69) | <0.001 |
| p,p'-DDT | Not detected | | Not detected | |  |
| HCB | 6.74 ± 3.60 | 6.36 (0.05, 33.04) | 6.49 ± 3.34 | 6.11 (0.05, 20.64) | 0.21 |
| β-HCH | 1.91 ± 1.52 | 1.72 (0.05, 12.83) | 0.09 ± 0.33 | 0.05 (0.05, 4.03) | <0.001 |
| γ-HCH | Not detected | | Not detected | |  |
|  | Brominated flame retardants (ng/g lipid weight) | | | |  |
| BDE 47 | 2.41 ± 3.38 | 1.46 (0.05, 20.19) | 0.92 ± 8.11 | 0.05 (0.05, 115.01) | <0.001 |
| BDE 99 | 0.29 ± 0.76 | 0.15 (0.15, 5.83) | 3.84 ± 47.09 | 0.15 (0.15, 667.98) | <0.001 |
| BDE 100 | 0.18 ± 0.17 | 0.15 (0.15, 1.53) | 0.88 ± 8.98 | 0.15 (0.15, 127.48) | <0.001 |
| BDE 153 | Not detected | | Not detected | |  |
| BDE 209 | Not detected | | Not detected | |  |
| α-HBCD | Not detected | | Not detected | |  |
| TBBPA | Not detected | | Not detected | |  |
|  | Per- and polyfluoroalkylated substances (ng/mL plasma) | | | |  |
| PFHxS | 0.07 ± 0.04 | 0.06 (0.01, 0.35) | 0.09 ± 0.06 | 0.08 (0.01, 0.51) | <0.001 |
| PFOS | 1.09 ± 0.44 | 1.02 (0.39, 3.55) | 0.88 ± 0.56 | 0.74 (0.22, 3.54) | <0.001 |
| PFBA | 0.02 ± 0.01 | 0.02 (0.01, 0.12) | 0.06 ± 0.54 | 0.01 (0.01, 7.64) | 0.004 |
| PFHxA | 0.006 ± 0.003 | 0.005 (0.005, 0.027) | 0.005 ± 0.001 | 0.005 (0.005, 0.013) | 0.004 |
| PFHpA | 0.014 ± 0.013 | 0.014 (0.005, 0.156) | 0.011 ± 0.012 | 0.005 (0.005, 0.148) | <0.001 |
| PFOA | 0.39 ± 0.22 | 0.36 (0.05, 1.78) | 0.33 ± 0.18 | 0.28 (0.06, 1.37) | <0.001 |
| PFNA | 0.14 ± 0.13 | 0.12 (0.02, 1.33) | 0.06 ± 0.04 | 0.05 (0.01, 0.24) | <0.001 |
| PFDA | 0.05 ± 0.07 | 0.04 (0.01, 0.82) | 0.03 ± 0.02 | 0.02 (0.01, 0.12) | <0.001 |
| PFUdA | 0.02 ± 0.02 | 0.02 (0.1, 0.2) | 0.02 ± 0.01 | 0.01 (0.01, 0.11) | <0.001 |
| PFDoA | 0.02 ± 0.04 | 0.01 (0.01, 0.54) | 0.01 ± 0.01 | 0.01 (0.01, 0.04) | <0.001 |
| PFTrDA | 0.009 ± 0.01 | 0.005 (0.005, 0.107) | 0.008 ± 0.007 | 0.005 (0.005, 0.045) | 0.82 |
|  | Monohydroxylated PAH metabolites (µg/g creatinine) | | | |  |
| Σ OH-NAP | 4.956 ± 6.66 | 3.46 (0.64, 67.22) | 6.51 ± 6.20 | 4.76 (0.35, 44.83) | <0.001 |
| 2-OH-FLUO | 0.07 ± 0.04 | 0.06 (0.01, 0.35) | 0.23 ± 0.14 | 0.20 (0.02, 1.14) | <0.001 |
| Σ OH-PHEN | 0.33 ± 0.32 | 0.25 (0,08, 2.67) | 0.98 ± 0.67 | 0.94 (0.06, 4.94) | <0.001 |
| 1-OH–PYR | 0.03 ± 0.03 | 0.02 (0.00, 0.20) | 0.04 ± 0.05 | 0.02 (0.00, 0.33) | 0.58 |

| **Table S4** Comparison of medicament administrations in mothers between localities | | | |
| --- | --- | --- | --- |
|  | Ceske Budejovice (N=147) | Karvina (N=201) |  |
|  | Variable (N, %) | | P |
|  | Prostaglandins | |  |
| Yes | 14 (9.52%) | 55 (27.36%) | <0.001 |
|  | Oxytocin | |  |
| Yes | 146 (99.32%) | 190 (94.53%) | 0.006 |
|  | Analgesics | |  |
| Yes | 60 (40.82%) | 9 (4.48%) | <0.001 |
|  | Spasmolytic | |  |
| Yes | 7 (4.76%) | 55 (27.36%) | <0.001 |

| **Table S5** Comparison of frequency maternal delivery factors between localities | | | |
| --- | --- | --- | --- |
|  | Ceske Budejovice (N=147) | Karvina (N=201) |  |
|  | Variable (N, %) – Z test | | P |
|  | Maternal long-term diagnosis (maternal questionnaire) | |  |
| Without | 112 (74.83%) | 130 (64.68%) | 0.04 |
| Thyroid | 15 (10.20%) | 10 (4.98%) | 0.08 |
| Allergy/asthma | 10 (6.80%) | 35 (17.91%) | 0.001 |
| DM | 2 (1.36%) | 4 (1.99%) | 0.65 |
| Others | 10 (6.80%) | 23 (10.45%) | 0.22 |
|  | Pregnancy diagnosis (maternal questionnaire) | |  |
| Without | 112 (76.19%) | 155 (77.11%) | 0.84 |
| Gestational DM | 10 (6.80%) | 22 (10.95%) | 0.17 |
| Other inflammation | 16 (10.88%) | 7 (8.46%) | 0.45 |
| Others | 9 (6.12%) | 7 (3.48%) | 0.26 |
|  | Maternal clinical risk factors (medical questionnaire) | |  |
| Without | 67 (45.58%) | 120 (59.70%) | 0.009 |
| Chronical | 4 (2.72%) | 4 (1.99%) | 0.66 |
| Acute pregnancy | 22 (14.97%) | 28 (13.93%) | 0.79 |
| Gilbert syndrome | 8 (5.44%) | 15 (7.46%) | 0.44 |
| Others | 46 (31.29%) | 34 (16.92%) | 0.002 |
|  | Type of delivery (medical questionnaire) | |  |
| Vaginal | 80 (54.42%) | 142 (70.65%) | 0.002 |
| CS | 66 (44.90%) | 55 (27.36%) | <0.001 |
| VEX | 1 (0.68%) | 4 (1.99%) | 0.27 |
|  | Anesthesia (medical questionnaire) | |  |
| Yes | 124 (84.35%) | 131 (65.17%) | <0.001 |
| No | 23 (15.65%) | 70 (34.83%) | <0.001 |
|  | Delivery procedures (medical questionnaire) | |  |
| Without | 80 (54.42%) | 138 (69.00%) | 0.006 |
| Pharmacological intervention | 14 (9.52%) | 8 (4.00%) | 0.05 |
| Induced delivery | 36 (24.49%) | 48 (24.00%) | 0.91 |
| Others | 17 (11.56%) | 6 (3.00%) | 0.003 |
|  | Delivery complications (medical questionnaire) | |  |
| Without | 118 (80.27%) | 168 (83.58%) | 0.43 |
| Blood loss > 500ml | 4 (2.72%) | 2 (1%) | 0.25 |
| Cloudy amniotic fluid | 9 (6.12%) | 10 (4.98%) | 0.65 |
| Potential fetal hypoxia | 6 (4.08%) | 14 (6.97%) | 0.23 |
| Others | 10 (6.80%) | 7 (3.48%) | 0.17 |
